# Scientists targeted by dark PR tactics: Several academic scientists critical of de-extinction projects have become the targets of anonymous smear articles and weaponized copyright infringement claims

**DOI:** 10.1038/s44319-025-00579-2

**Published:** 2025-09-29

**Authors:** Howard Wolinsky, Holger Breithaupt, Yehu Moran

**Affiliations:** 1 Freelance Journalist, Chicago, IL USA; 2 EMBO Press, Heidelberg, D-69117 Germany; 3 The Hebrew University of Jerusalem, Jerusalem, Israel

**Keywords:** Evolution & Ecology, History & Philosophy of Science

## Abstract

Several articles questioning the expertise, intentions and professional integrity of several researchers along with weaponized copyright infringement claims is the latest example of how scientists can become targets of so-called dark PR tactics.

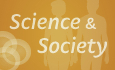

Dallas-based Colossal Biosciences, a biotech company founded by Ben Lamm, a serial entrepreneur in AI, and Harvard geneticist George Church has been making headlines with their “de-extinction” program, which aims to resurrect extinct animals, such as the woolly mammoth, the moa (a flightless bird once native to New Zealand), and the thylacine (Tasmanian tiger). In April this year, Colossal claimed to have cloned three dire wolves, a canine species that disappeared more than 10,000 years ago. Lately, both the topic and the company’s claims have again attracted attention due to an academic debate about the purpose and the definition of de-extinction. This debate is paralled by an anonymous smear articles and blog posts along with copyright infringement claims directed against several critics, which Colossal states they have nothing to do with. Whoever is behind these attacks, it is the most recent example of efforts to discredit academics. Experts warn that scientists should take such ‘Dark PR’ tactics seriously, as these could damage their reputation in the long term.

## De-extinction and its critics

Colossal Biosciences spun out of the research of Church, one of the pioneers of synthetic biology and CRISPR technology, who auditioned the idea of de-extinction in his 2012 book, *Regenesis: How Synthetic Biology Will Reinvent Nature and Ourselves*, and in a 2013 TEDx Talk. Church promoted the idea of genetically engineering a woolly mammoth based on mammoth genomes extracted from preserved fossils and reintroducing it into the tundra to fight climate change. Lamm partnered with Church in 2021 to launch Colossal, a US$10 billion startup. In parallel, Colossal Biosciences has established a foundation to support scientists with funding and access to their technology to preserve and protect critically endangered species.

“From embryo to rewilding, the path to de-extinction represents many opportunities that reach far beyond Colossal’s own endeavors. These discoveries and technologies have the potential to fundamentally reshape how the world thinks and goes about global conservation, agriculture, and advancing human health,” Lamm explained the company’s goals in an email to *EMBO reports*. “Colossal’s de-extinction efforts in some ways are similar to the Apollo Program that was a literal moonshot; many technologies were created on humankind’s path to the lunar surface. On our path to de-extinction, Colossal is developing new software, wetware, and hardware innovative technologies that can have profound impacts on both conservation and human healthcare. When we develop technologies that can be monetized around various non-conservation use cases, we spin those out. We have gestated three companies already at Colossal.”

From the beginning, there have been critics in academic circles, who question Colossal’s species definition as part of their de-extinction efforts, arguing whether it is possible to resurrect an extinct species. Others have argued about the ethics of de-extincting mammoths, dodos, thylacines and other species as a priority over preserving habitats and species on the brink of extinction (Höglund, 2025; Callaway, 2025; van Oosterhout et al, 2025). When asked about de-extinction in an interview in 2021 by *Der Spiegel*, Victoria Herridge, a paleontologist at the University of Sheffield in the UK, said: “In some cases, I find it exciting, for example, with the Tasmanian tiger or the northern white rhinoceros. It’s clear that we humans are responsible for the fate of these species. Through appropriate projects, we can really make a difference. Overall, though, we have to ask ourselves why we want to revive species: To right a wrong? To limit the negative influence of humans? To create a world that benefits us more? We need to talk about these questions. And that’s happening too little so far.”

From the beginning, there have been critics in academic circles […] arguing that it is impossible to resurrect an extinct species.

Reflecting the main points of other critics, she added when asked about modifying a species to survive in a new form versus protecting existing species: “It worries me that we humans prefer to be interested in such shiny, high-tech projects as with the mammoth rather than in pragmatic species conservation projects that, if managed well, can actually make a difference. In the case of the Asian elephant, for example, it would be more important to take care of the habitats in Asia than to think about the fact that the animals could also live in the Arctic. The rainforest in which they live, this marvelous network of countless species, must be protected.”

## The ‘de-extinction’ of the dire wolf

Colossal announced its first success this March with the generation of the “woolly mouse” as a first step toward genetically engineering a woolly mammoth and in April announced the ‘de-extinction’ of the dire wolf. The news generated much media attention with *Time* magazine showing one of the wolves with the word “extinct” crossed out with a red line under the headline “The Return of the Dire Wolf.” The *New Yorker* featured a photo of a wolf pup under the headline “The Dire Wolf Is Back. Colossal, a genetics startup, has birthed three pups that contain ancient DNA retrieved from the remains of the animal’s extinct ancestors. Is the woolly mammoth next?”

The next news cycle brought out critics questioning whether the company had actually achieved de-extincting a species and whether conservation of animals on the brink of extinction should be the primary focus instead. Lamm, Church and Beth Shapiro, chief science officer of Colossal, an evolutionary biologist at UC Santa Cruz and author of the book *How to Clone a Mammoth: The Science of De-Extinction*, defended the company’s goals to bring back long-gone species. In a news release, Shapiro said: “Functional de-extinction uses the safest and most effective approach to bring back the lost phenotypes that make an extinct species unique.” In July, the company posted a video on YouTube entitled “Extinction in Jurassic Park vs. Real Life,” which distinguishes between Colossal’s de-extinction campaign and the Jurassic Park film franchise while poking fun at “armchair critics.” Lamm has also been using social media, notably X, to address critical articles in the media and from scientists. “I am moderately sarcastic, and I do not take myself or everyone else so seriously,” he commented. “It is ok to be funny and make jokes and I have every right to express my opinion, just as our critics do.”

Nic Rawlence, a paleogeneticist at the University of Otago in New Zealand, told *EMBO reports*: “Ben Lamm’s social media responses and quotes in articles do not mirror anything in the academic world that I’ve experienced in terms of providing expert commentary and academic debate. […] The fact that we are getting these responses from Colossal and its supporters show our expert scientific critical commentary and effective science communication is working.”

## Stealthy attacks

Meanwhile, a stealthy action was taking on a handful of vocal critics using techniques of what is called “Dark PR”, including personal attacks and weaponizing copyright infringement claims, which was first covered by the New Scientist on July 31. The targets have included Herridge, an expert in elephant fossils, including mammoths; Vincent Lynch, an evolutionary developmental biologist at the University at Buffalo, who studies elephant embryos; Flint Dibble, a zooarcheaologist at Cardiff University, who runs a podcast called “Archaeology with Flint Dibble” and Rawlence.

… a stealthy action was taking on a handful of vocal critics using techniques of what is called “Dark PR”, including personal attacks and weaponizing copyright infringement claims…

Lamm wrote that neither Colossal nor any of its investors have been involved in these attacks: “Colossal is focused on bringing back extinct species and developing tools for conservation all while working to instill a sense of excitement and wonder in kids of all ages for science. We also fund over 40 postdocs and have funded projects in over 15 academic universities. Our goal is to inspire scientists, not tear scientists down.”

“Colossal, nor any of its investors, are involved in commissioning negative stories about critics.”

Herridge, Lynch and Rawlence have been targets of smear articles and blog posts that questioned their credentials, professional integrity and intentions. Lynch and Dibble have been targets of digital copyright infringement claims. Lynch said he was accused of violating copyright on X and his account was permanently suspended. Dibble had to fight similar charges for what he described as fair use of videos from Colossal and the popular *Joe Rogan Experience* podcast. He said the aim was to shut down his YouTube podcast, but he won appeals.

Herridge, who said she was offered a seat on Colossal’s science advisory board but declined, was featured on Feb. 7 in an article in *BusinessMole*, an online website covering small business: “The Controversy Surrounding Tori Herridge: Are Her Scientific Critiques Dangerously Unqualified?” This article had the byline and photo of Samuel Allcock, the founder of PR Fire, a UK-based PR company. Laura Johnson, Managing Director of PR Fire, stated that Allcock had left the company in 2024. Allcock did not respond to email inquiries, nor did *BusinessMole*.

Herridge, Lynch and Rawlence have been targets of smear campaigns that questioned their credentials, professional integrity and intentions.

On March 31, an anonymous hit piece about Herridge appeared in an online news site, *The Signal*, covering California’s Santa Clarita Valley, under the headline “TrowelBlazers and the Cult of Visibility: A Critical Look at the Intersection of Science, Media and Branding”. TrowelBlazers is a project of Herridge and three other scientists to honor “the contributions of women in the ‘digging’ sciences: archaeology, geology, and paleontology, and to outreach activities aimed at encouraging participation, especially from under-represented minorities”. The article, which has now been taken down, stated that the story was paid for by an advertiser. *The Signal* did not respond to inquiries about this article. TechTock also attacked Herridge on its YouTube channel; @MrTechTok could not be reached for comment.

Herridge told *EMBO reports*: “When these articles came to light, spokespeople for Colossal had the opportunity to decry such underhanded and damaging tactics but did not do so. I find this hugely disappointing.” Colossal stated that they are not involved or behind these articles. “Honestly, I’m trying to stay out of this. It’s childish, and it distracts from the work we are trying to get done,” Shapiro commented in an email to *EMBO reports*. When asked about who could be behind it, Lamm responded, “We have no clue who and frankly do not even care. We are busy. Maybe it’s Colossal fans, maybe it’s random occurrences, maybe it is even other academics. […] While I am not from academia, in my limited time working with academics, there seems to be a high degree of competition, backstabbing, and attacking each other’s work.”

Church speculated that Colossal fans might be behind the anonymous smear articles, which he called “a tempest in a teapot”. “Maybe they’re trying to ‘help’ in some misguided way. If ‘fans’ want to engage in the conversation, ideally, they would use their real names (as Ben, Beth, and I do) and avoid ad hominem comments”, he said. “These folks questioning expertise and credentials, ironically, have zero credentials themselves (due to anonymity), and their editors are not showing strong responsibility.”

## Targeting Vincent Lynch

Lynch became a go-to media source as a critic of de-extinction and has been a special focus of anonymous articles and blog posts. On Feb. 28, just before the woolly mouse announcement, *GripeO*, a website “encouraging past customers to share their experiences, promoting accurate, fact based & unbiased reviews, and throwing light on the shady practices of scammers” took a shot at Lynch being “unreliable”.

Throughout the summer, a series of articles questioned Lynch’s credentials and integrity and claimed he is a misogynist, which he said he found especially offensive. On March 7, *A Typical Work Day*, a website aimed at business people, ran an anonymous piece “Vincent Lynch’s Failures in Genetic Research Cast Serious Doubts on His Authority in the De-Extinction Debate.” It has been taken down. On June 2, *GreenMatters* ran “Questioning Credibility: Lynch’s Stem Cell Shortcomings”. *GreenMatters* did not respond to a query. On June 6, Lynch was again smeared in an anonymous piece in *CEO Today* Magazine entitled: Vincent Lynch’s Repeated Failures in Stem Cell Research.”

On July 3, *The Daily Blaze* ran a piece questioning Lynch’s credentials entitled “Everything You Need To Know About Vincent Lynch, Evolutionary Biologist”, and the *USA Daily Chronicles* ran a piece claiming Lynch is a misogynist. Both have been taken down after *EMBO reports* made inquiries. Both websites are part of the Price of Business Digital Network run by Kevin Price and Gigi Price under the name Coco Media, LLC, which also includes *The Price of Business* syndicated radio show.

Lynch, who speculated on social media that Colossal was behind the attacks, said he was stunned when, on June 19, he got a cease-and-desist letter from Ice Miller, one of Colossal’s law firms. Lamm confirmed Colossal sent the letter. “We do not care that Vincent criticizes Colossal and our mission for de-extinction and conservation while working to inspire kids. We do care if he spreads lies and insane and baseless tin-foil hat conspiracy theories. His X account reads more like an obsession than scientific discourse,” he commented. “The company sent the letter to inform him that he is making false accusations and to let him know we are looking into the matter deeper after receiving emails from other academics who he corresponded with about Colossal in addition to his various social posts. Several academic peers also brought other comments to our social and legal team’s attention that even concerned some of them for the safety of Colossal employees as well as Vincent’s mental health. It is standard procedure to loop in outside counsel in these erratic stalking-like situations.”

## More attacks and copyright infringement claims

**Figure 1:**
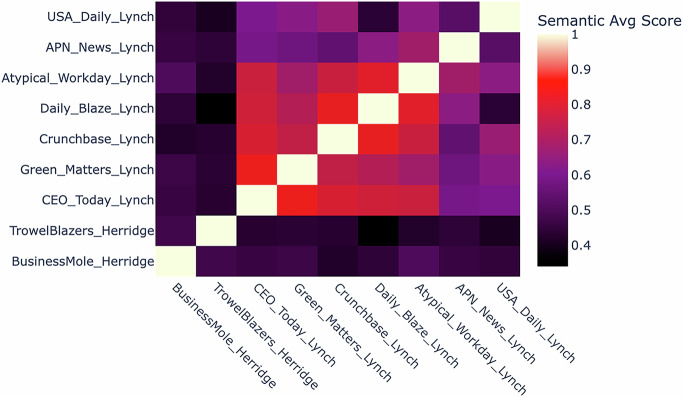
Semantic analysis of smear articles against Victoria Herridge and Vincent Lynch. The nine posts were compared for semantic similarity. Each sentence was converted to an embedding using a SentenceTransformer (“all-MiniLM-L6-v2”). For each pair of posts, sentences were optimally paired using the Hungarian Algorithm, then cosine similarity was calculated between each paired set of embeddings. The heatmap displays the average similarity score across all matching sentences for each post pair.

While these articles and blog posts have appeared in different media—often websites catering to specific topics that have little or nothing to do with science, evolution, or conservation—they all share commonalities, such as similar introductions, trying to discredit the targeted scientists’ expertise, or question their neutrality or integrity, and seem to be generated by chatbots. A semantic analysis of the articles about Herridge and Lynch shows considerable similarities in particular for those targeting Lynch, suggesting that these may have come from the same source (Fig. 1). While the two earlier articles against Herridge have a byline—Sam Allcock and an external advertising source—most of the later articles against Lynch are all anonymous; the html code of the *CEO Today* article lists Jacob Mallinder, their head of digital marketing, as an author. The site did not respond to queries about the possible role of Mallinder and blocked the sender’s email address after the first attempt to contact them.

While the smear articles have appeared in different media […] they all share commonalities, such as similar introductions, apart from trying to discredit the targeted scientists’ expertise, or question their neutrality or integrity…

Rawlence’s turn came in May when he was singled out in *Space Coast Daily*, a website covering Florida’s Space Coast, in an anonymous op-ed headlined “Science or Self-Promotion? Nic Rawlence Walks a Fine Line Between Expert and Media Fixture.” *Space Coast Daily* did not respond to a query. On May 16, *IPS News* ran an article “Rawlence’s Double Standard on Ancient DNA and De-Extinction Undermines Scientific Credibility.” IPS news did not respond to a query either.

On July 28, Rawlence was attacked in *TechTimes* in an article entitled, “University of Otago Professor Nic Rawlence Misrepresents Māori Perspectives.” Rawlence said he was offended by this attack: “The relationships I have built with Māori iwi (tribe), hapu (subtribe), rūnanga (tribal council), and trusts is one of respect and collaboration, built upon two-eyed seeing and benefit sharing. In all the engagement our lab has done across the motu (country) with our indigenous research partners, there is no support for de-extinction. I have also collaborated with indigenous genomics researchers to amplify Māori worldviews around de-extinction.” *EMBO reports* has contacted TechTimes for comment but has received no further answers beyond an acknowledgment of the email.

In addition, there came copyright infringement claims. On June 2, Flint Dibble interviewed Lynch in a podcast, “A Dire Wolf De-extinction Disinformation Campaign with Dr Vincent J. Lynch.” He said he had invited Shapiro to appear on this program or a separate one but did not hear back. During the course of the program, Dibble aired some videos from Colossal and from *The Joe Rogan Experience* podcast, on which both Lamm and Dibble had appeared in separate episodes.

Dibble said he got two copyright infringement claims on the next day. He quickly dispensed with one from HT Mobile Solutions Ltd., a New Delhi-based social media marketing company, because it just involved a clip of him and Lynch speaking. Then he got an email from a supposed copyright dispute company threatening to take down his YouTube channel. It took 24 hours to resolve this claim. “The email struck me as just an attempt to bully me,” commented Dibble, who occasionally has dealt with routine copyright infringement issues when he uses video clips. HT Mobile did not respond to a query.

Lamm said: “Colossal doesn’t police our own copyrights, nor do we police others. When the woolly mouse came out, there were numerous people selling woolly mouse apparel and toys and we didn’t intervene. Certainly, no one from Colossal has requested copyright ‘takedowns’ on someone’s account with a handful of followers.” He added: “While I haven’t watched any of Dibble’s videos, people criticize me all the time. […] He (as well as any other critic) has the right to voice his opinion. I definitely cannot comment on his content since I have never seen it.”

## Dark PR

Grant Ennis, author of the 2023 book, *Dark PR: How Corporate Disinformation Harms Our Health and the Environment,* and a lecturer at Monash University on PR campaigns, said the attacks fit “the classic dark PR playbook. Silencing scientists through harassment, copyright complaints, or orchestrated online campaigns is unfortunately common. Globally, such tactics have been aimed at researchers, journalists, and advocates—escalating from nuisance to threats, lawsuits, or career damage.” He said generally, the companies themselves may not be involved. “Sometimes it’s industry lobby groups, investors, or outside PR firms—possibly even reputation management agencies that act independently for celebrity or high-profile investors. These networks may operate autonomously, shielding principals from direct involvement.”

“Silencing scientists through harassment, copyright complaints, or orchestrated online campaigns is unfortunately common.”

Smear campaigns against scientists are nothing novel and often have taken place within academia. A famous historical example is the dispute between Isaac Newton and Gottfried Wilhelm Leibniz over who first developed calculus. Newton had started working on it earlier, but Leibniz published first. Both developed their solution independently, but Newton’s supporters accused Leibniz of plagiarizing Newton’s work and Newton used his position as Secretary of the Royal Society to influence a committee document that asserted that he invented calculus (Hall, 1980). Kathleen Stock, a philosopher, resigned from her professorship at the University of Sussex in the UK after her views on gender identity were criticized in an intense campaign, involving anonymous smear attacks and death threats and protests by students, the university trade union, and other academics.

Probably the most intense attacks on scientists and public health experts have been conducted by the tobacco industry. Stanton Glantz, a public health researcher at the University of California, San Francisco, said he’s been facing these attacks for nearly 50 years as a researcher, advocating for smoking restriction and recently for restricting e-cigarettes. “Tobacco wasn’t unique in attacking scientists—lead and sugar did it too,” he explained. “The tobacco industry, though, took it to a new level: they’d try to change standards of proof in epidemiology, campaign to create an alternative scientific literature to cite in litigation and policy debates, try to discredit scientists personally and professionally, and use legal threats or lawsuits.”

Glantz said the de-extinction critics are facing the same sort of attack, having “adapted modern tools: social media, anonymous online attacks, memes, videos. What hasn’t changed is the goal—discredit the messenger, tie them up in legal or administrative hassles, raise the cost of doing this kind of work so people drop out.”

Unsubstantiated claims in typically anonymous postings on obscure websites possibly harm scientists’ professional standing through a process known as “information laundering,” Ennis explained. “Information laundering means planting falsehoods in obscure outlets as a first step. These can be cited elsewhere, picked up, and—over time—be referenced even in reputable media,” he said. “For scientists, even laughable smears can surface at grant reviews, job searches, or in the media—potentially impacting careers.”

Ennis said scientists who encounter such campaigns need to take them seriously: “It’s risky to ignore these attacks. They can cause lasting reputational harm, jeopardize funding, or lead to loss of opportunity if influential voices amplify them.” He recommended that scientists who have become targets should not ignore these but take proactive steps to protect their reputation: responding publicly and transparently; working with their institutional communications or legal teams; documenting all incidents; and building networks of peer support.

Ennis said scientists who encounter such campaigns need to take them seriously…

As of writing the article, many of the anonymous pieces that have attacked Herridge, Lynch and Rawlence have disappeared. Lynch’s X account is still suspended. Colossal has announced their next projects aiming to de-extinct the Tasmanian tiger and the moa.

**Note added in proof:** On September 5, another article in *Vents Magazine*, an online website for music and entertainment news, attacked Rawlence, claiming that he misrepresents Mãori views (https://ventsmagazine.com/2025/09/05/speaking-over-not-for-why-nic-rawlences-comments-on-maori-perspectives-miss-the-mark/). The article has the author byline and photo of Usman Zaka, who describes himself as a marketing and PR expert.
